# Rule-Mining for the Early Prediction of Chronic Kidney Disease Based on Metabolomics and Multi-Source Data

**DOI:** 10.1371/journal.pone.0166905

**Published:** 2016-11-18

**Authors:** Margaux Luck, Gildas Bertho, Mathilde Bateson, Alexandre Karras, Anastasia Yartseva, Eric Thervet, Cecilia Damon, Nicolas Pallet

**Affiliations:** 1 Paris Descartes University, Paris, France; 2 Hypercube Institute, Paris, France; 3 Renal Division, Georges Pompidou European Hospital, Paris, France; The University of Tokyo, JAPAN

## Abstract

^1^H Nuclear Magnetic Resonance (NMR)-based metabolic profiling is very promising for the diagnostic of the stages of chronic kidney disease (CKD). Because of the high dimension of NMR spectra datasets and the complex mixture of metabolites in biological samples, the identification of discriminant biomarkers of a disease is challenging. None of the widely used chemometric methods in NMR metabolomics performs a local exhaustive exploration of the data. We developed a descriptive and easily understandable approach that searches for discriminant local phenomena using an original exhaustive rule-mining algorithm in order to predict two groups of patients: 1) patients having low to mild CKD stages with no renal failure and 2) patients having moderate to established CKD stages with renal failure. Our predictive algorithm explores the m-dimensional variable space to capture the local overdensities of the two groups of patients under the form of easily interpretable rules. Afterwards, a L2-penalized logistic regression on the discriminant rules was used to build predictive models of the CKD stages. We explored a complex multi-source dataset that included the clinical, demographic, clinical chemistry, renal pathology and urine metabolomic data of a cohort of 110 patients. Given this multi-source dataset and the complex nature of metabolomic data, we analyzed 1- and 2-dimensional rules in order to integrate the information carried by the interactions between the variables. The results indicated that our local algorithm is a valuable analytical method for the precise characterization of multivariate CKD stage profiles and as efficient as the classical global model using chi2 variable section with an approximately 70% of good classification level. The resulting predictive models predominantly identify urinary metabolites (such as 3-hydroxyisovalerate, carnitine, citrate, dimethylsulfone, creatinine and N-methylnicotinamide) as relevant variables indicating that CKD significantly affects the urinary metabolome. In addition, the simple knowledge of the concentration of urinary metabolites classifies the CKD stage of the patients correctly.

## Introduction

Chronic kidney disease (CKD) affects millions of persons worldwide and constitutes a major public health problem and an economic challenge [1]. Understanding the molecular profile of CKD is a key challenge for the early disease diagnosis and the development and optimization of preventive and therapeutic strategies. Because CKD does not cause symptoms until it is in an advanced stage, the development of analytical tools to detect CKD in its earlier stages is of great interest. Therefore, integrated biological approaches for understanding how phenotypic pattern changes define the pathophysiology and the severity of kidney disease conditions are urgently needed.

“Metabolomics is a global biochemical approach for biomarker discovery and provides insight into metabolic profiling across a wide range of biochemical pathways in response to a perturbation (disease, drugs, and toxins)” [2]. As, the endogenous metabolites are the final product of interactions between gene expression, protein expression, and the cellular environment; it provides information that is both complementary to these other molecular domains. Moreover, it also reflects exogenous sources of variation (diet, environment and microbiome) which is meaningful for the study of complex human disease [3].

Nuclear magnetic resonance (NMR), which uses the magnetic properties of a given atomic nuclei to assess the abundance of metabolites in a biological sample, has been the primary technique used since it requires little sample preparation, and provides absolute quantities, and is highly reproducible. However, its sensitivity is limited to high-concentration metabolites. More recently, GC-MS or LC-MS have become more widely used due to its greater sensitivity based on chromatographic separation but failed to identify some metabolites due to uncertainty in the estimation of metabolites’ mass-to-charge ratios. “Continued advances in NMR spectroscopic hardware and increased parallel application of analytical platforms such as Ultra-performance liquid chromatography (UPLC) [4, 5] coupled with quadrupole time-of-flight mass spectrometry (UPLC-QTOF/MS) will enable even wider coverage of the metabolome” [2].

Metabolomics using NMR and MS techniques is proving to be a promising technology for the identification of early biochemical changes, potential biomarkers and underlying disease mechanisms associated with kidney disease [6, 7, 8]. Zhao et al, in [2], focus on the identification of metabolomic biomarkers of drug-induced renal toxicity. Indeed, many toxicants (antibiotics, anticancer agents, immunosuppressive drugs and heavy metals) may have an impact on the renal function leading to nephrotoxicity. Therefore, the identification of these specific biomarkers provides meaningful information for the development of more biocompatible therapeutics. In line with the growing interest of renal metabolomics, numerous studies have been published, focusing on uremic toxins characterization, acute kidney injury, diabetic nephropathy, autosomal dominant polycystic kidney disease, and renal cell carcinoma and kidney transplatation [9].

Chemometric methods classically used in ^1^H NMR metabolomics for high-dimensional classification problems combine a dimension reduction step and a classification step [10, 11, 12, 13]. These methods perform a variable selection that assesses the global information carried by each variable and related to the target. We call them global variable selection. However, in biological processes, local phenomena generated by the heterogeneity of the populations, the complex composition of biological data and huge local interactions between variables play a major role. Consequently, the use of variable selection searching for local phenomena appears to be a valuable approach to go beyond the limited global variable selection. Recently, local analysis with Random Forests approaches have been applied for NMR prediction [12]. However, Random Forests only explore random parts of the variable space and are difficult to interpret. We recently proposed an exhaustive local predictive method that first extracted local variables with a rule-mining algorithm and then built a local predictive model of CKD stages with a L2-penalized logistic regression [14].

The complex and noisy nature of the metabolomic data, composed of a mixture of molecules with overlaps of several metabolites and some metabolites appearing at different spectra frequency, involve discriminant interactions between variables. Moreover, the clinical and biological characteristics of the patients (such as age, sex, weight, diet or chemistry information) might affect the spectral composition of the samples as well as the outcome of interest adding potential discriminant sources of data [15]. Hence, considering the nature of the metabolomic data and the possible usefulness of additional sources of data, we propose to extend our previous work by adding 2D rules (i.e., two distinct conditions of variables) in order to integrate the interactions between the variables. In addition, we considered in this study other available sources of data. By this process, we aimed to accomplish the following: (1) to verify whether the multidimensional rules enhanced the models in terms of predictive power and medical knowledge and (2) to determine which were the most relevant variables among the metabolomic and clinical data. Therefore, we could provide indications into the way in which the urine metabolome and other clinico-biological data characterize the CKD stages.

We used a multi-source dataset of clinical data, demographics, clinical chemistry, renal pathology and urine metabolomic data as explanatory variables as an input for our algorithm. We wanted to test whether and how a combination of variables allows us to describe individuals with an estimated Glomerular Filtration Rate (eGFR) ≥ 60 ml/min/1.73 m^2^ corresponding to KDIGO CKD stages 1 and 2 (no renal failure) and individuals with an eGFR < 60 ml/min/1.73 m^2^ corresponding to KDIGO CKD stages 3 to 5 (renal failure). We first constructed the local 1D rules and 1&2D rules models (i.e., resp. with and without variable interactions) solely on metabolomic data on the one hand and on the multi-source data on the other. Then, we compared these local models with a classical global model performing a chi2 feature selection followed by a L2-penalized logistic regression.

Our results indicate that our local predictive models were as powerful as the global model and they provided more precise medical information. Moreover, from a biological standpoint, the 1&2D model goes further than the 1D model in providing more useful information into how the interactions between features from the urine metabolome, and other biological data, characterize the CKD stages. Finally, we observed that the majority of the predictive variables were urinary metabolites identified by ^1^H NMR, indicating that CKD significantly affects the urinary metabolome.

## Results

### Comparative evaluation of global and local models on metabolomics and multi-source data

We first aimed at evaluating and comparing the three models (global, local 1D, local 1&2D) on metabolomics and multi-source data. In Table 1, we compared the prediction F1-score, the statistical significance (pvalue), the complexity and the stability of the different models (see Methods).

**Table 1:** Evaluation of the global, local 1D and local 1&2D models.

**Metabolomics dataset**					
model	F1-score	complexity	pvalue	C (freq)	stability
global	0.73±0.09	10	0.008	1.0 (29)	0.56±0.16
local 1D	0.70±0.1	19	0.01	1.0 (48)	0.43± 0.14
local 1&2D	0.69±0.1	339	0.01	1.0 (50)	0.19±0.13
**Multi-source dataset**					
global	0.71±0.1	10	0.006	0.1 (31)/1.0 (30)	54±0.15
local 1D	0.69±0.1	19	0.01	1.0 (48)	0.42±0.14
local 1&2D	0.67±0.1	360	0.01	1.0 (49)	0.18±0.13

On both types of datasets, metabolomics (urine ^1^H NMR metabolomic data alone) and multi-source (clinical data, demographics, clinical chemistry, renal pathology, urine metabolomic data), for each of the three models (global, local 1D, local 1&2D), the table reports the F1-score (mean±sd), the average complexity computed over 100 runs, the p-value estimated with the permutation test, the most frequent C regularization coefficient(s) and its occurrence frequency in parentheses (freq) across the 100 runs as well as the stability of the model defined as the common percentage of selected variables between runs (mean±sd).

All the models (global, local 1D and local 1&2D) were able to discriminate patients with an eGFR ≥ 60 ml/min/1.73m^2^ from patients with an eGFR < 60 ml/min/1.73m^2^ with an equally significant p-value (=1 x 10^−2^ with a permutation test). One of the most common optimal values of C (i.e., the regularization coefficient of the L2-penalized logistic regression) was 1.0 for all the models. This result indicates that our variable selection step (see Methods) is efficient enough to achieve good performance with our classifier without the use of smooth solutions requiring a strong regularization. Given the initial number of variables, we observed that our local variable selection is particularly stringent leading to low complexity models.

The stability measures were lower for the local models than for the global model. A perfect overlap between 1D rules and even more between 2D rules is likely strongly disturbed by several phenomena as follows: 1) the low number of patients leading to small differences in the dataset splits (train/test) might have induced strong perturbations in the rules’ generation, 2) the nature of the metabolomic data including the overlap of some metabolites in the ^1^H NMR spectra and that two variables could interact together because they could correspond to buckets (i.e., parts of the metabolomic spectrum, see Methods) that fit the same molecule, or that belong to the same function or biological pathway.

Finally, no differences were observed between the F1-scores of the models constructed with the metabolomic data and with the multi-source data, be they global, local 1D or local 1&2D models. However, the local models should provide more meaningful and more precise information because of the insights given by the rules.

The performance of our local models was as significant as the performance of the global model, and they provided richer information useful for further medical interpretation.

### Metabolomic profiles reflect CKD severity

We investigated the variables selected by the models to identify the urine metabolomic profile predictive of CKD.

**Fig 1:**
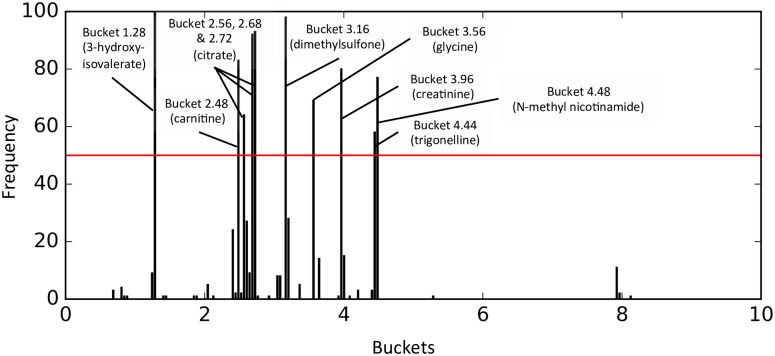
Buckets’ frequency over the 100 metabolomic global models. This figure shows the frequency (y axis) of each bucket selected over the 100 global models built on the metabolomic data (x axis). The red horizontal line corresponds to a frequency threshold of 50. Buckets with a frequency higher than this threshold are labeled with their corresponding metabolites.

For the global model, 42 buckets out of the 184 were present at least once (Fig 1). The most commonly identified buckets (present in at least 50% of the models) were 1.28 (3-hydroxyisovalerate); 2.48 (carnitine); 2.56, 2.68 and 2.72 (citrate); 3.16 (dimethylsulfone); 3.56 (glycine); 3.96 (creatinine); 4.44 (trigonelline), and 4.48 (N-methyl nicotinamide). The corresponding metabolites were identify manually using the Human Metabolome Database (HMDB), http://www.hmdb.ca/ [16, 17].

**Fig 2:**
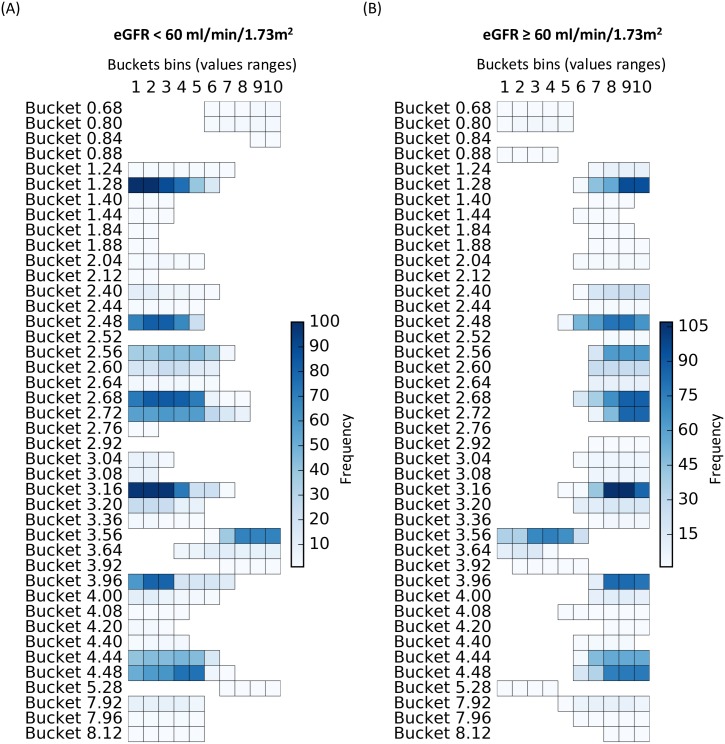
Buckets’ frequency over the 100 metabolomic local 1D models. Each horizontal segment corresponds to a 1D rule characterized by its bucket’s name, covered values ranges (i.e., buckets bins which could be interpreted as relative concentration) and frequency (color scale). The more robust the rule is, the darker it will be. (A) shows the rules corresponding to the subgroup of patients with an eGFR <60 ml/min/1.73m^2^ and conversely, (B) shows the rules corresponding to the subgroup of patients with an eGFR ≥ 60 ml/min/1.73m^2^.

For the local 1D model, at least one significant rule was selected for each one of the 42 pre-selected global variables (Buckets) for at least one of the two subgroups of patients (eGFR < 60 ml/min/1.73m^2^ or eGFR ≥ 60 ml/min/1.73m^2^) (Fig 2). The rules characterized by the same bucket for both eGFR < 60 and ≥ 60 ml/min/1.73m^2^ are generally disjoint (i.e., covered disjoint sub-regions of the bucket space). In rare cases, an overlap occurs between rules of the two modalities (i.e., the group of patients), which might reflect a bias in the discretization process. The mainly disjoint rules indicate that individuals with an eGFR < 60 ml/min/1.73m^2^ were specifically characterized by a low concentration of 3-hydroxyisovalerate, carnitine, citrate, dimethylsulfone, creatinine, trigonelline and N-methyl nicotinamide and high concentrations of glycine, the inverse being observed for individuals with eGFR ≥ 60 ml/min/1.73m^2^. These findings reinforce the idea that our rule-mining algorithm allows the classification of groups of patients based on the local trends (i.e., range of values) of some variables.

**Fig 3:**
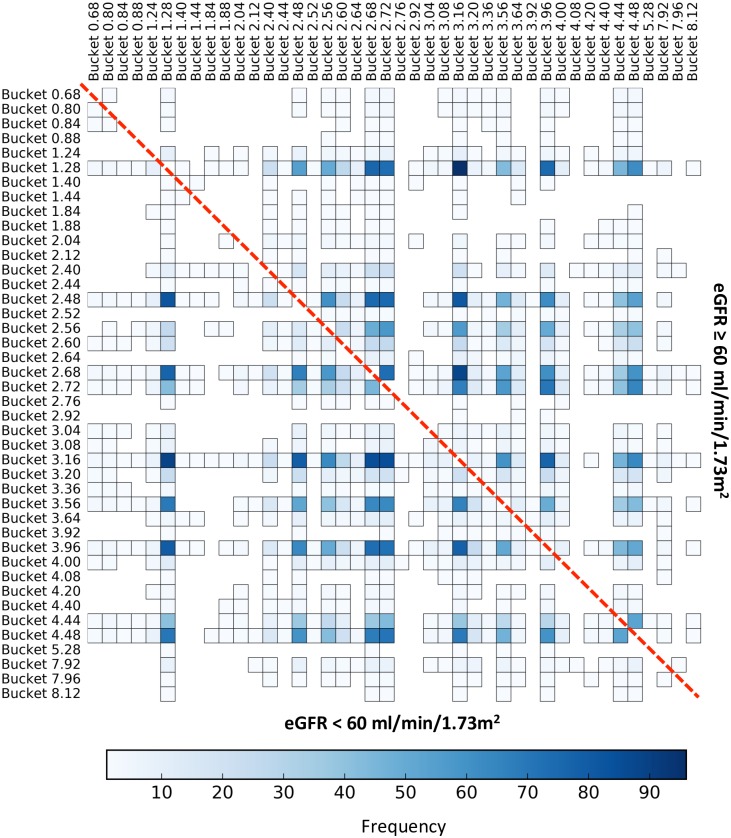
Frequency of the bucket interaction over the 100 metabolomic local 1&2D models. This figure shows the frequency heatmap of the interaction (color scale) of each bucket pair over the 100 local 1&2D models. The more robust the interaction is, the darker it will be. The interactions corresponding to the subgroup of patients with an eGFR < 60 ml/min/1.73m^2^ are displayed in the lower triangle and conversely, the interactions corresponding to the subgroup of patients with an eGFR ≥ 60 ml/min/1.73m^2^ are displayed in the upper triangle. The limit of the two triangles is represented by the red dashed-line.

**Fig 4:**
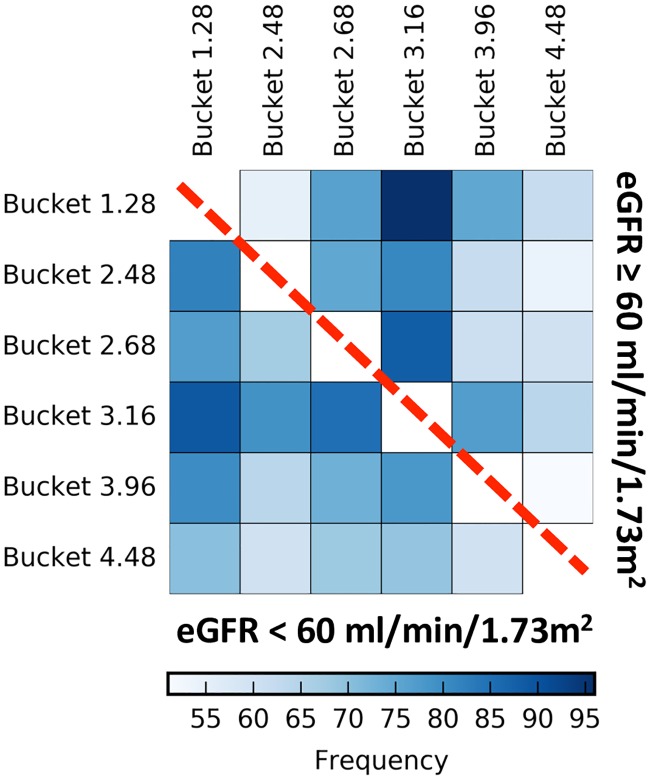
Frequency of the cluster of buckets present in at least 50% of the local 1&2D models. This figure shows the frequency heatmap of the interactions (color scale) of each bucket pair of the cluster over the 100 local 1&2D models. The more robust the interaction is, the darker it will be. The interactions corresponding to the subgroup of patients with an eGFR < 60 ml/min/1.73m^2^ are displayed in the lower triangle and conversely, the interactions corresponding to the subgroup of patients with an eGFR ≥ 60 ml/min/1.73m^2^ are displayed in the upper triangle. The limit of the two triangles is represented by the red dashed-line.

**Fig 5:**
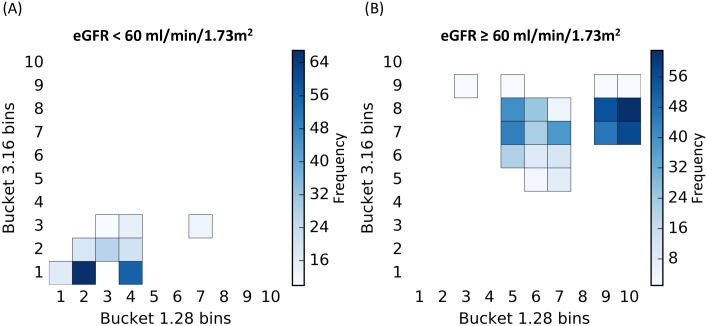
2D rules implicated in the interaction between buckets 1.28 (3-hydroxyisovalerate) and 3.16 (dimethylsulfone). The 2D rules are represented with their variable conditions, i.e., ranges of buckets bins for the buckets 1.28 (3-hydroxyisovalerate) (x-axis) and 3.16 (dimethylsulfone) (y-axis) and their frequency over the 100 models (color scale). The most frequent the bucket bin is covered by the 2D rules, the darker it will be. (A) shows the rules corresponding to the subgroup of patients with an eGFR < 60 ml/min/1.73m^2^ and conversely, (B) shows the rules corresponding to the subgroup of patients with an eGFR ≥ 60 ml/min/1.73m^2^.

Our local 1&2D model allowed us to identify a cluster of variables that frequently interacted, that is in at least 50% of our models (Fig 3). These couples of variables typically appeared for both subgroups of patients. This cluster includes 6 buckets (Fig 4) corresponding to known metabolites, such as 1.28 (3-hydroxyisovalerate), 2.48 (carnitine), 2.68 (citrate), 3.16 (dimethylsulfone), 3.96 (creatinine) and 4.48 (N-methylnicotinamide). The local interactions between buckets 1.28 (3-hydroxyisovalerate) and 3.16 (dimethylsulfone), which are the most frequent in models are represented in the Fig 5. These results indicate that patients with an eGFR < 60 ml/min/1.73m^2^ have at the same time low concentrations of 3-hydroxyisovalerate and dimethylsulfone, with a mirror profile for patients with an eGFR ≥ 60 ml/min/1.73m^2^.

It is important to understand that, despite having similar predictive power, our local model has an enhanced explanatory power providing more meaningful and easily interpreted information than that provided by the global model. Moreover, the set of learned rules could be derived for further validation of predictive or prognostic value.

### Multi-source profile of CKD severity

**Fig 6:**
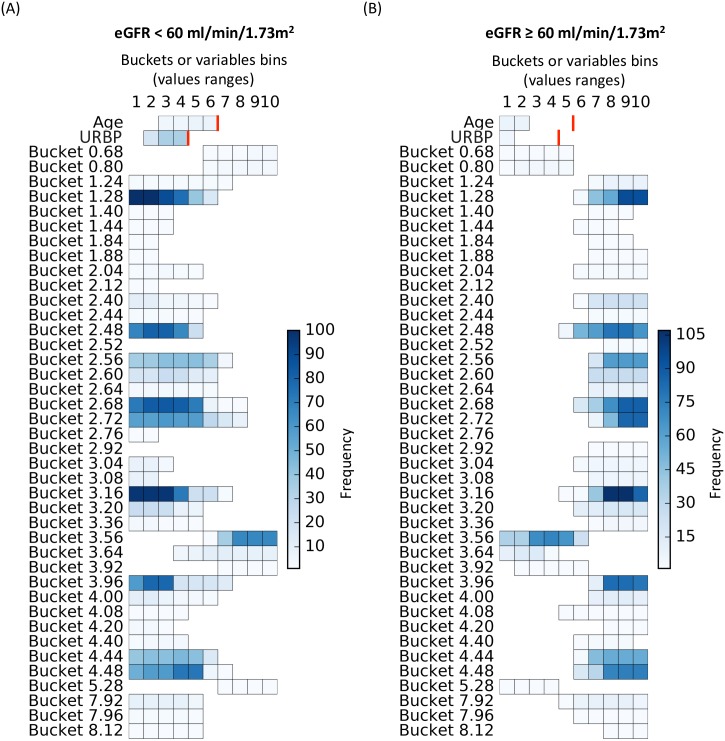
Variables’ frequency over the 100 multi-source local 1D models. Each horizontal segment corresponds to a 1D rule characterized by its variable condition: the variable’s name and the set of covered values or bins (n.b., ranges of bucket’s bins could be interpreted as relative concentrations). The color scale reflects the frequency of the variables’ values covered by the rules. As the two non-bucket variables were discretized according to the clinical relevance for the urinary retinol binding protein concentration (URBP) and the variable distribution for the Age, we indicated with a red line the corresponding upper bin of these two variables. The more robust the rule, the darker it will be. (A) shows the rules corresponding to the subgroup of patients with an eGFR < 60 ml/min/1.73m^2^ and conversely, (B) shows the rules corresponding to the subgroup of patients with an eGFR ≥ 60 ml/min/1.73m^2^.

The models computed on the multi-source dataset (i.e., clinical, demographic, clinical chemistry, renal pathology and urine metabolomic data) were in most cases composed of the same variables as those from the models applied solely to the metabolomics dataset. Among the variables selected by the global model, only the following two variables were not metabolomic data: the age and the urinary retinol binding protein concentration. The local 1D model revealed that the patients with an eGFR < 60 ml/min/1.73m^2^ were older and had a high concentration of urinary retinol binding protein and, conversely for individuals with an eGFR ≥ 60 ml/min/1.73m^2^ (Fig 6).

**Fig 7:**
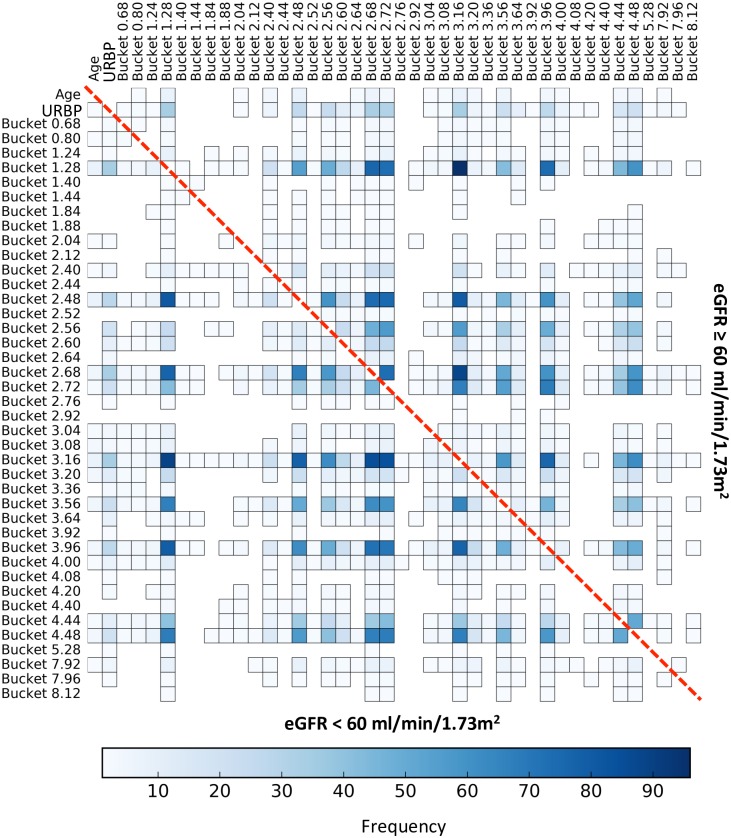
Frequency of the bucket interaction over the 100 multi-source local 1&2D models. This figure shows the frequency heatmap (color scale) of the interactions of each multi-source variable pair over the 100 local 1&2D models. The more robust the interaction, the darker it will be. The interactions corresponding to the subgroup of patients with an eGFR < 60 ml/min/1.73m^2^ are displayed in the lower triangle and conversely, the interactions corresponding to the subgroup of patients with an eGFR ≥ 60 ml/min/1.73m^2^ are displayed in the upper triangle. The limit of the two triangles is represented by the red dashed-line.

Additionally, the local 1&2D models (Fig 7) highlight that these variables interacted for individuals with an eGFR < 60 ml/min/1.73m^2^ and with the buckets belonging to the cluster identified in the metabolomic local 1&2D models (Figs 3 and 4) for both the groups of individuals < 60 and ≥ 60 ml/min/1.73m^2^. However, these two non-metabolomic variables were present in less than 35% of the 100 global models, explaining the similar performances obtained on both metabolomic and multi-source datasets with the three type of models.

Simply knowing the concentration of urinary metabolites allows us to correctly identify the eGFR class of individuals.

## Discussion

The principle of rule-mining approach is to explore some parts of the variable space and to generate rules difining sets of variables with their ranges of values predictive of subpopulations having a high density for the outcome of interest. Our rule-mining algorithm has the particularity of being able to explore all possible combinations of subspaces of variables without any prior assumptions. In this study, we have made the proof of concept of the usefulness and feasibility of generating rules to address a major clinical issue: the detection of CKD in its early stage. An important advantage of this approach is to produce pathophysiological hypotheses as well as biomarkers.

Briefly, from a methodological standpoint, our local predictive approach is as powerful as the global one, provides information on the distribution of the variables’ values according to the outcome of interest and uses multi-source data interactions (2D). The higher complexity of our local models allows for more interpretable and meaningful solutions. However, the minimization part of the rule-mining algorithm might be revisited to remove many redundancies within the final set of rules. Revisiting this part of the algorithm should allow us to take into account rules with more than 2 dimensions. Furthermore, an increase in complexity is hugely time-consuming, leads to a higher risk of false discoveries, and could be difficult to interpret.

From a biological standpoint, our local model goes further than the global one providing important clues into how the urine metabolome, and other biological data, characterize the early stage of CKD. The local 1&2D model is very interesting because it extracted and highlighted a cluster of six metabolomic variables that interact together with each other to predict the two CKD stages. Through all the resulting models, we observed that the majority of the predictive variables were metabolites indicating that the urinary metabolome is affected in the evolution of CKD. In addition, we found that metabolites are better predictors of CKD stages than the classical parameters, such as fibrosis or proteinuria. We found a reduction in citrate levels in CKD patients, which could be related to well-known metabolic disorders including conditions that lower the renal tubular pH or intracellular pH, such as the metabolic acidosis present in CKD patients [18]. As the hospitalized individuals of our studied cohort have been on a strict diet excluding for instance sodas, it is unlikely that variations of citrate concentration result from feed intake. Conversely, we found a decreased concentration of dimethylsulfone in the urine of individuals having an altered renal function. Dimethylsulfone has been identified as an uremic toxin with optimized metabolomic ^1^H NMR approaches [19]. Moreover, the presence of dimethylsulfone likely produced by the gut has been described many years ago for its antioxidant and anti-inflammatory properties, at least in vitro [20, 21, 22]. A decreased urinary concentration of dimethylsulfone in individuals with an altered renal function might be a consequence of a reduced clearance without compensatory tubular secretion. Additionally, we have identified low concentrations of trigonelline in urine samples of patients with an altered renal function. This finding is reasonable because trigonelline is an alkaloid produced by the metabolism of niacin (vitamin B3), which is excreted in the urine and might protect against renal oxidative stress and apoptotic cell death and slow the progression of diabetic nephropathy [23]. To conclude, we have identified a set of urinary metabolites as good and non-invasive predictors of the eGFR levels, indicating that the urinary metabolome is a potential source for non-invasive markers of early renal dysfunction function.

The repartition of patients into two groups, one with eGFR < 60 ml/min/1.73 m^2^ and the other with eGFR ≥ 60 ml/min/1.73 m^2^, has been defined according to the major variables of the clinical picture of renal disease. The small size of the cohort, and in particular the relatively small proportion of patients with mild CKD, may be also somewhat a limitation of the study for the application of our models to a more general population. Despite the few discriminating metabolites identified by our models compared to the whole urinary metabolome [24], our results are consistent with the metabolomic signatures of patients with advanced-stage CKD, found in recent studies [25], [26]. Moreover, our approach provided clues to the pathophysiology of CKD, as it has been exemplified with the role of mitochondrial dysfunction in the course of diabetic nephropathy unraveled with urine metabolome [27]. It is worth noting that these relevant results have been found notwithstanding a coarse ^1^H NMR spectra pre-processing. In terms of data, several ways of improving this study will be to refine the CKD stages, increase the number of subjects and review the H NMR spectra pre-processing.

The usefulness of our rule-mining algorithm in medicine is straightforward because it allows us to identify non-invasive biomarkers of ongoing renal injury and it could facilitate the generation of hypotheses about the CKD pathogenesis. Indeed, searching for local overdensities in an m-dimensional space, explained by easily interpretable rules, is thus seemingly ideal for generating hypotheses for large datasets to unravel the inherent complexity in biological systems. This proof of concept study will pave the way for further applications of our rule-mining algorithm in renal medicine, for example, for the prediction of the response to a therapy. The application of this tool theoretically targets all disciplines of life science. In addition, our approach could be used for the joint analysis of omics data (i.e., peptidomics, proteomics and genomics data). Consequently, we envision that improvements in personalized medecine will require data mining tools such as our algorithm that could be implemented in Clinical Data Warehouses [28], [29], [30].

## Methods

### Dataset

#### Recruitment and sample collection

The Paris Descartes University ethics committee (the Patients Protection Committee) approved the study design. Before any medical consultation, the patients provided written consent and the cohort was then anonymized. Between 2013 and 2014, 110 consecutive CKD patients referred to the Nephrology Department of the Georges Pompidou European Hospital (Paris, France) for a kidney biopsy were included in the study (see Table 2 for the baseline characteristics of the study cohort). The selection criteria for performing the patient biopsy were as follow: an estimated glomerular filtration rate (eGFR) < 60ml/min and/or proteinuria > 0.5 g/l and/or the presence of hematuria. Urine and blood samples were collected, at the time of biopsy (mid-morning) and stored at -80°C until routine clinical chemistry and metabolomic analyses. All the individuals were hospitalized for at least 12 hours before the urine sampling. Additional control of the patients’ diets was not necessary as the samples were taken in a hospital that already imposed a specific diet. The day of the biopsy, the following multi-source patient data were recorded using an information-based data warehouse [31]: clinical (age, sex, body mass index, arterial hypertension), demographic (ethnicity), clinical chemistry (urinary total protein, urinary albumin, urinary retinol binding protein, urinary *β*2-microglobulin, urinary *α*1-microglobulin, urinary transferrin, serum creatinine), renal pathology (interstitial fibrosis extent) and urine metabolomic (^1^H NMR spectra) data. These multi-source data correspond to the explanatory variables (input variables) in our analysis except for the serum creatinine (used to calculate the target). As a prerequisite to our analysis, missing values of a variable are replaced with the median of that variable and all of the explanatory variables were discretized according to the clinical relevance or variable distribution. The objective of this monitoring is to identify individual profiles predictive of CKD stages.

**Table 2:** Baseline characteristics of the study cohort (n = 110).

**Demographics**	
Age (years)	55±18
Male gender	65 (65)
Caucasian patients	71 (64)
Body Mass Index (kg/m2)	25.9±4.6
Hypertension	74 (67)
Hyperlipidemia	24 (22)
Diabetes	11 (10)
Cancer	16 (15)
**Etiology of CKD**	
FSGS/minimal changes disease	15 (14)
ANCA-mediated vasculitidis	10 (9)
IgA nephropathy	9 (8.5)
Primary Glomerulonephritis	9 (8.5)
Lupus	7 (6.5)
Diabetes	7 (6)
Tubule-interstitial nephropathy	7 (6)
Hypertensive nephropathy	7 (6)
Non specific	7 (6)
Myeloma cast nephropathy	6.6 (5.5)
Other	24
**Medications**	
CEI/ARA2	47 (43)
Diuretics	18 (17)
Beta blockers	13 (12)
Calcium Channel Inhibitors	25 (23)
Others	4.5 (4)
**Clinical Chemistry**	
Serum creatinine (mg/dl)	2.08±1.45
eGFR (ml/min/1.73m2)	44.6±33
Proteinuria (g/l)	2.3±2.7
Albuminuria (g/l)	1.3±2.4

Continuous variables are expressed as the mean±sem. Categorical variables are expressed as n (%).

#### Clinical chemistry analyses

The serum and urine clinical chemistry measurements were performed in the Clinical Chemistry Department of the European Georges Pompidou Hospital the day of the kidney biopsy. The urinary total protein was measured with a pyrogallol red-molybdate complex at 600/800 nm absorbance (urinary CSF protein assay, Beckman Coulter), and the urinary albumin was measured by an immunoturbidimetry (DIAgAM assay, Beckman Coulter), both using a Beckman Coulter AU680 analyzer (Beckman Coulter, Brea, CA, USA). The urinary levels of *α*1-microglobulin, *β*2-microglobulin, transferrin, and retinol binding protein were measured with kits from Siemens using a Siemens Dabe Behring BN II Nephelometer Analyzer (Siemens HeathCare GmbH, Erlangen, Germany). The urine samples were alkalinized prior to measuring the *β*2-microglobulin levels. The serum creatinine was measured using a colorimetric assay (modified kinetic Jaffe method) on a Beckman Coulter DXC analyzer (Beckman Coulter, Brea, CA, USA). The eGFR was calculated using the modification of diet in renal disease (MDRD) equation [32]. Two stages of CKD severity were defined for our analysis corresponding to the following 2 groups of patients: 24 patients with an eGFR ≥ 60 ml/min/1.73m2 corresponding to low and mild CKD and 86 patients with an eGFR < 60 ml/min/1.73m2 corresponding to CKD stages ranging from moderate to established kidney failure according to the KDIGO CKD Work Group [33]. These two CKD severity stages correspond to the target variable (output) in our analysis.

#### Measurement of the renal pathology (interstitial fibrosis extent)

The cortex areas of the biopsy were analyzed, whereas the medulla was excluded from the analysis. For the analysis, an image of the cortical section of each kidney biopsy was captured using the Hamamatsu slide scanner (Hamamatzu Protonics, Iwata City, Japan), Nanozoomer with an objective 20x, NA = 0.75, and a Hamamatsu 3-CCD camera. The quantification of the extent of interstitial fibrosis was performed using Massons trichrome-stained kidney sections with color segmentation image analysis software [34]. The green colored areas characteristic of interstitial fibrosis were measured whereas the areas corresponding to the renal capsule, tubular basement membranes, glomeruli, and vessel were automatically excluded from the analysis.

#### ^1^H NMR data acquisition

The urine samples were prepared with chemical product from Sigma (Sigma Aldrich, Saint Quentin Fallavier, France) to obtain a final volume of 600 *μ*L (400 *μ*L of urine; 160 *μ*L of 200 mM phosphate buffer at pH 7.4, 1 mM of TSP -Trimethyl silyl propionate of sodium salt as the NMR chemical shift reference-, 6 mM of NaN_3_; 40 *μ*L D_2_O). Urine ^1^H NMR spectra were measured at 300K on a Bruker Avance II 500 MHz spectrometer (Brucker Biospin GmbH, Rheinstetten, Germany) equipped with a SampleXpress automation sample changer and a standard 5 mm BBI probe with Z-gradient. The spectra acquisition was based on a 1D Nuclear Overhauser Effect Spectroscopy (NOESY) pulse sequence with presaturation for water suppression. The parameters used for the pulse sequence were as follows: a relaxation delay of 1 s, a mixing time of 100 ms, an acquisition time of 1.36 s and a 90 degree pulse length of 8 *μ*s. Data points (32K) were collected during 64 scans with a spectral width of 20 ppm. The preprocessing of the urine ^1^H NMR spectra was performed with MestReNova 8.0 software. A line-broadening factor of 0.3 Hz prior to Fourier transformation was applied. The spectra were then phased, baseline corrected, and referenced to TSP. Each NMR spectrum (0.16 to 9.00 ppm) was reduced by an equidistant binning method with a bin width of 0.04 ppm to limit misalignment problems. The spectral regions corresponding to urea (5.45 to 6.48 ppm) and water (4.48 to 4.96 ppm) were deleted to remove variability because of the suppression of water. Then, an integral normalization (typically a constant integral of 100) was performed on the remaining 184 bins. The resulting matrix was then normalized with a pareto scaling giving an NMR spectra dataset of 110 individuals and 184 buckets. Finally, we discretized the 184 buckets with a 10 quantile-based binning.

### Supervised analysis

This work extends the study presented at the ICMLA conference [14] by considering 2D rules and multi-source data and revisiting the evaluation procedure. We built three predictive models (global, local 1D and local 1&2D) integrating as previously a supervised variable selection step and a classification step. They differ by the variable selection step using the chi2 test with a p-value threshold of 0.05 corrected with Bonferroni for the global predictive model and our rule mining algorithm applied to the variables selected in the global model for the local models. The local 1D model selects local variables from the 1D rules (considering one variable condition) whereas the local 1&2D model selects, in addition, couples of variables from 2D rules (considering two variables conditions). For the classification step, we used the L2-regularized logistic regression leading to linear and smooth solutions. These models were first only applied on the metabolomic data and then on the multi-source data. The data used for the supervised analysis are available in Supporting Information (see S1 Dataset). Our rule-mining algorithm has been developed in-house in python and we used the famous sklearn python library for the chi2 test and the logistic regression.

#### Rule-mining algorithm

**Fig 8:**
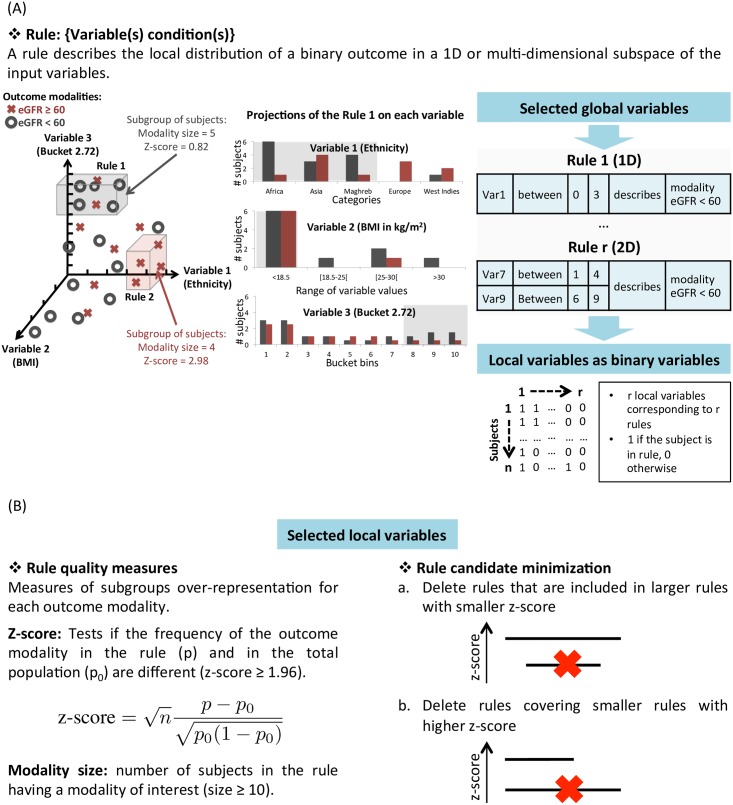
Rule-mining algorithm. (A) shows the representation of a rule in the multi-dimensional variable space (left), projected on each variable (middle) and as binary variable spanning over all the subjects (right). (B) shows the selection of the candidate rules proceeding in the following two steps: thresholding on two rule quality measures (left) and a minimization procedure to remove redundant rules (right).

A complete description of our rule-mining algorithm is detailed in Algorithm 1 [14]. We illustrated the process of this algorithm in Fig 8. It will exhaustively explore the multi-dimensional variable space in order to extract regions (i.e. hypercube) having over-concentration of a class of subjects (here, one of the two CKD severity stages). We recall that these regions may be represented under the form of a rule:

Rule:{Variable(s)condition(s)}

where variable(s) condition(s) correspond to one value or a range/set of values of a given variable observed in the dataset. For example, 1D and 2D rules might be: 1D rule: {variable: ‘bucket 2.14’;condition: bucket bins 1 to 4} and 2D rule: {variable 1: ‘sex’;condition: ‘women’ AND variable 2: ‘bucket 3.16’;condition: bucket bin 6}.

We represented these rules as binary variable spanning all the subjects, designated here as local variables, and used as input to the classifier (Fig 8A). To identify the most discriminative rules, we used the following two quality measures: the rule modality size and the z-score. The rule modality size is the number of subjects in the rule having one of the two modalities of interest. We selected rules with a rule modality size ≥ 10 to ensure robust and generalizable rules. We selected rules with a z-score≥1.96 corresponding to a significant difference between the proportion of the outcome modality in the rule and in the total population. Based on these candidate rules, we removed the redundant rules, i.e., those sharing the same feature(s) and condition(s) and having worse quality measures. This step of our algorithm is called minimization (Fig 8B). We can summarize the minimization procedure in two steps. The first step consisted in deleting rules that are included in larger rules with smaller z-score and the second step consisted in deleting rules covering smaller rules with higher z-score.

**Algorithm 1** Supervised rule mining (see S1 Fig)

**Input:**

- a tuple of variables (feature(s), target): 1 (resp. 2) feature(s) for 1D (resp. 2D) rules

- two thresholds for the rule quality measures: rule modality size ≥ 10 and z-score ≥ 1.96

**Output:**

- a set of significant rules for each modality

**Step 1: Exhaustive rule generation and selection**

a. For each feature

 Construct the candidate rules from all the possible combinations of ranges/sets of values of one or two variables

b. For each target modality

 For each candidate rule

  Compute quality measures: modality size, z-score

  if modality size ≥ 10 and z-score ≥ 1.96

   keep the rule

**Step 2: Rule candidate minimization**

a. Delete rules that are included in larger rules with smaller z-score.

b. Delete rules covering smaller rules with higher z-score.

#### Evaluation

Because we had a small number of subjects, we first splitted the dataset into a train (80%) and a test (20%) sets in which the percentages of samples of each class were preserved. We performed this step 100 times to avoid being dependent on a particular random selection of a test set, which could lead to biased results. For each of these 100 models, we optimized the C parameter of the L2-regularized logistic regression with an inner cross-validation composed of 10 runs of 2-fold cross-validation to ensure a sufficient number of individuals in the less represented class. We computed the average F1 score over 100 splits, fixing C to be the most frequent value optimized in the inner cross-validation. Then, we assessed the statistical significance of our evaluation procedure using a permutation test of 1000 runs. This test evaluated whether our models, built on the original dataset, were significantly better than any other models obtained by randomly permuting the patients’ output of the original dataset (eGFR < 60 ml/min/1.73m^2^ or eGFR ≥ 60 ml/min/1.73m^2^). Finally, we assessed the stability of our models over the 100 runs by calculating the common percentage of selected variables between runs. This was calculated using the Jaccard similarity coefficient, which measures the similarity between finite sets of selected variables, and is defined as the size of the intersection divided by the size of the union of the sets of selected variables. For the global models, we considered the original global variables selected by the chi2 test. For the local models, we considered the selected local variables, which are the variables conditions.

## Supporting Information

S1 Dataset Data used for the supervised analysis. In sheet 1 is provided the discretized data used for the supervised analysis and in sheet 2 the related metadata.
(XLS)

S1 Fig Steps of the supervised rule mining algorithm: an example with the 1D rule case. (A) Step 1: Exhaustive rule generation and selection. A segment represents a 1D rule defined by its feature condition FC (i.e., range of variable bins on x-axis). Selected rules called candidates rules (circled) have, for one of the two modalities a rule modality size ≥ 10 and a z-score ≥ 1.96. (B) Step 2: Rule candidate minimization. Two rule elimination stages are applied: first (step 2.a.), rules included in larger rules with smaller z-score are crossed out with continuous line and secondly (step 2.b.) rules covering smaller rules with higher z-score are crossed out with dashed line.
(TIF)
