# Supplementary figures and images for: Rule-Mining for the Early Prediction of Chronic Kidney Disease Based on Metabolomics and Multi-Source Data
Source: PLoS One. 2016 Nov 18;11(11):e0166905. doi: 10.1371/journal.pone.0166905 (PMC5115883; doi:10.1371/journal.pone.0166905)

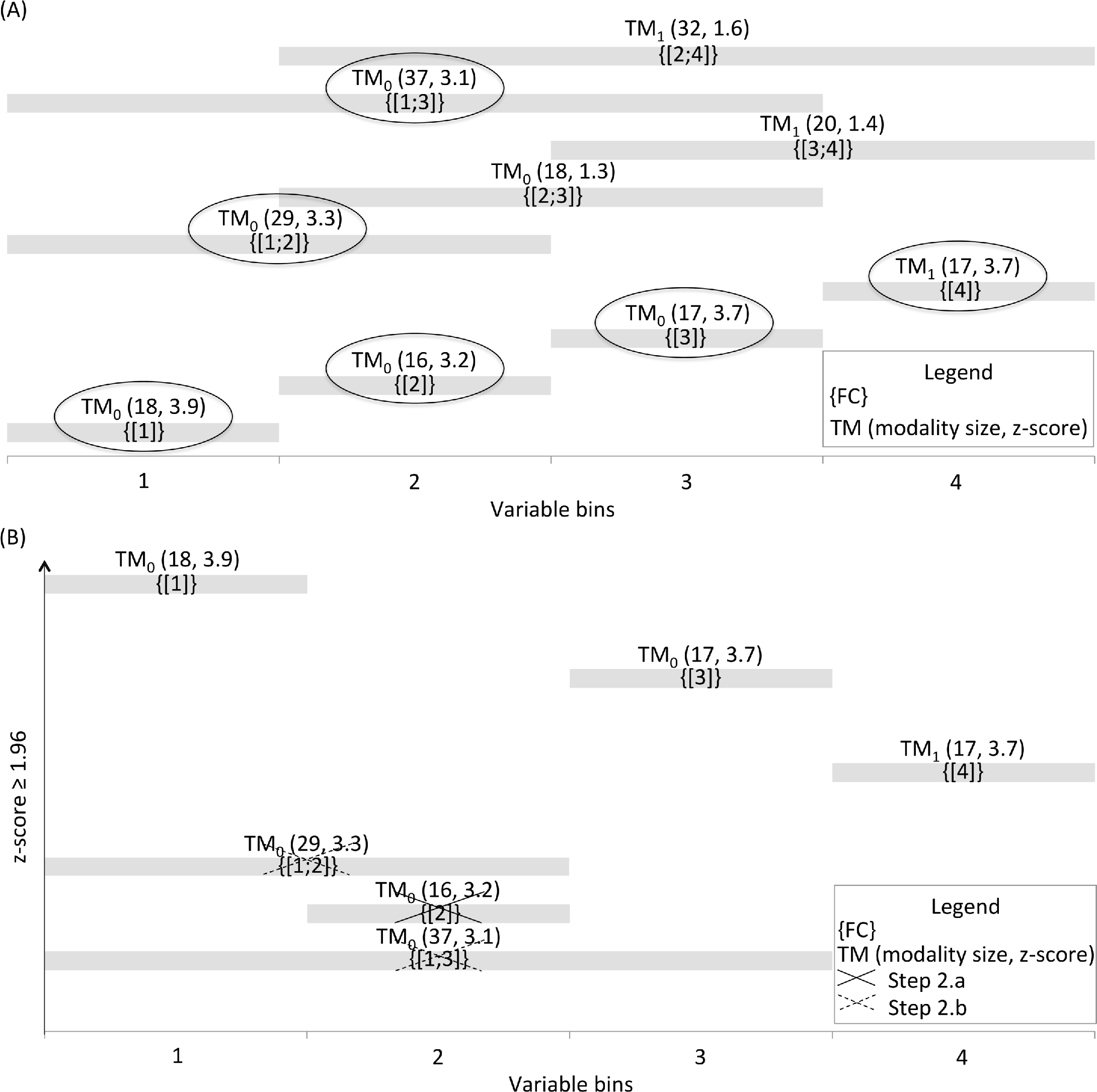

Supplement: S1 Fig — (A) Step 1: Exhaustive rule generation and selection. A segment represents a 1D rule defined by its feature condition FC (i.e., range of variable bins on x-axis). Selected rules called candidates rules (circled) have, for one of the two modalities a rule modality size ≥ 10 and a z-score ≥ 1.96. (B) Step 2: Rule candidate minimization. Two rule elimination stages are applied: first (step 2.a.), rules included in larger rules with smaller z-score are crossed out with continuous line and secondly (step 2.b.) rules covering smaller rules with higher z-score are crossed out with dashed line. (TIF) [file pone.0166905.s002.tif]
